# Supplementary material for: A Likelihood Approach for Real-Time Calibration of Stochastic Compartmental Epidemic Models
Source: PLoS Comput Biol. 2017 Jan 17;13(1):e1005257. doi: 10.1371/journal.pcbi.1005257 (PMC5240920; doi:10.1371/journal.pcbi.1005257)
Supplement: S1 File — (TAR.GZ) [file pcbi.1005257.s014.tar.gz › HSPH_Online-SI-Revision/IPoi09/n100-extreme/n100-extreme_BENCH2_summary.pdf]

| peak -8 | 3w spec | 3w cum | 5w spec | 5w cum | end | 3w spec LOG | 3w cum LOG | 5w spec LOG | 5w cum LOG | end LOG |
|---|---|---|---|---|---|---|---|---|---|---|
| mean | 208.6 | 222.7 | 87.2 | 121.1 | 23. | 0.7 | 0.7 | 0.7 | 0.7 | 0.4 |
| median | 119.6 | 138.8 | 71.6 | 90.3 | 13. | 0.5 | 0.5 | 0.4 | 0.5 | 0.1 |
| quantile .1 | 60.3 | 63.1 | 30.2 | 54.4 | 4. | 0.3 | 0.4 | 0.1 | 0.3 | 0. |
| quantile .9 | 333.8 | 379.2 | 170.3 | 200.8 | 55.6 | 1.2 | 1.2 | 1.5 | 0.3 | 1.2 |
| 40 | 39 | 38 | 37 | 23 | | | | | | |

| peak -4 | 3w spec | 3w cum | 5w spec | 5w cum | end | 3w spec LOG | 3w cum LOG | 5w spec LOG | 5w cum LOG | end LOG |
|---|---|---|---|---|---|---|---|---|---|---|
| mean | 29.6 | 40.6 | 27.3 | 23.5 | 13.4 | 0.2 | 0.3 | 0.2 | 0.2 | 0.1 |
| median | 24.5 | 38.8 | 22.6 | 18.8 | 9.4 | 0.2 | 0.2 | 0.1 | 0.1 | 0. |
| quantile .1 | 15.7 | 26.9 | 14.4 | 13.3 | 3.1 | 0.1 | 0.1 | 0.1 | 0.1 | 0. |
| quantile .9 | 44.7 | 57.8 | 53.5 | 38.1 | 31. | 0.4 | 0.4 | 0.4 | 0.1 | 0.2 |
| 37 | 42 | 29 | 35 | 34 | | | | | | |

| peak | 3w spec | 3w cum | 5w spec | 5w cum | end | 3w spec LOG | 3w cum LOG | 5w spec LOG | 5w cum LOG | end LOG |
|---|---|---|---|---|---|---|---|---|---|---|
| mean | 37. | 24.2 | 68.6 | 28.5 | 36.3 | 0.2 | 0.1 | 0.2 | 0.1 | 0.2 |
| median | 34.8 | 21.6 | 49. | 28.1 | 34. | 0.1 | 0.1 | 0.2 | 0.1 | 0.1 |
| quantile .1 | 21. | 15.3 | 36.5 | 16.7 | 25.1 | 0.1 | 0.1 | 0.1 | 0.1 | 0.1 |
| quantile .9 | 50.8 | 38.1 | 99.4 | 43. | 48.5 | 0.3 | 0.2 | 0.4 | 0.1 | 0.3 |
| 47 | 49 | 38 | 47 | 46 | | | | | | |

| peak +4 | 3w spec | 3w cum | 5w spec | 5w cum | end | 3w spec LOG | 3w cum LOG | 5w spec LOG | 5w cum LOG | end LOG |
|---|---|---|---|---|---|---|---|---|---|---|
| mean | 131.8 | 91.8 | 253.5 | 102.6 | 116.2 | 0.3 | 0.2 | 0.4 | 0.3 | 0.3 |
| median | 62.2 | 53.9 | 81.8 | 56.3 | 63.1 | 0.2 | 0.2 | 0.3 | 0.2 | 0.2 |
| quantile .1 | 44.2 | 40.3 | 47.6 | 41.8 | 43.3 | 0.2 | 0.1 | 0.2 | 0.2 | 0.2 |
| quantile .9 | 299.9 | 204.8 | 459.1 | 229.4 | 244.5 | 0.5 | 0.4 | 0.7 | 0.2 | 0.5 |
| 35 | 39 | 32 | 38 | 36 | | | | | | |

| peak +8 | 3w spec | 3w cum | 5w spec | 5w cum | end | 3w spec LOG | 3w cum LOG | 5w spec LOG | 5w cum LOG | end LOG |
|---|---|---|---|---|---|---|---|---|---|---|
| mean | 298.9 | 222.7 | 376.6 | 239.3 | 253.4 | 0.4 | 0.3 | 0.4 | 0.4 | 0.4 |
| median | 98.2 | 92. | 108.5 | 94. | 105.2 | 0.3 | 0.3 | 0.3 | 0.3 | 0.3 |
| quantile .1 | 50.3 | 49.2 | 49.7 | 49.3 | 49.9 | 0.2 | 0.2 | 0.2 | 0.2 | 0.2 |
| quantile .9 | 496.2 | 335.3 | 690.9 | 360.6 | 402.8 | 0.7 | 0.6 | 0.8 | 0.2 | 0.6 |
| 27 | 28 | 24 | 28 | 26 | {} | {} | {} | {} | {} | {} |
